# Survey data on the quality of life of consumers fitted with osseointegrated fixation and bone-anchored limb prostheses provided by government organization

**DOI:** 10.1016/j.dib.2019.104536

**Published:** 2019-09-17

**Authors:** Laurent Frossard, Luciann Ferrada, Debra Berg

**Affiliations:** a Griffith University, Gold Coast, QLD, Australia; b University of the Sunshine Coast, Maroochydore, QLD, Australia; c Queensland University of Technology, Brisbane, QLD, Australia; d YourResearchProject Pty Ltd, Brisbane, QLD, Australia; e Queensland Artificial Limb Service, Brisbane, QLD, Australia

**Keywords:** Amputation, Artificial limb, Bone-anchored prosthesis, Direct skeletal attachment, Experience, Government, Osseointegrated implants, Osseointegration, Patient’ experience, Prosthesis, Quality of life, Satisfaction

## Abstract

The data in this paper are related to the research article entitled “Development of a government continuous quality improvement procedure for assessing the provision of bone anchored limb prosthesis: A process re-design descriptive study” (Frossard et al., Canadian Prosthetics & Orthotics Journal, 2018. 1(2). p. 1–14). This article contains quality of life data experienced by individuals before and after implantation of a press-fit or screw-type osseointegrated fixation when fitted with conventional socket-suspended and bone-anchored limb prosthesis, respectively. This specifically-designed survey was developed and administered by Queensland Artificial Limb Services (QALS), an Australian State government organization. It was an integrated part of QALS′ continuous quality improvement procedure for assessing the provision of bone-anchored prosthesis. A total of 12 out of the 65 consumers completed to the survey, giving a return rate of 18%. This benchmark information can contribute to inform the design of (A) other patients' experience surveys including those built-in governmental continuous quality improvement procedure as well as (B) clinical trials looking at the overall effects of surgical implantation of ossoeintegrated fixation on patients' quality of life. Online repository contains the files: https://data.mendeley.com/datasets/bkbxxmrhfh/1.

**Table:** Specifications Table

Subject area	Health service delivery
More specific subject area	Quality of life of individuals using lower limb prosthesis
Type of data	Table, graph
How data was acquired	Survey data
Data format	Raw and Analyzed
Experimental factors	A total of 65 consumers fitted with at least one osseointegrated fixation and bone-anchored limb prosthesis between 01/2011 and 06/2019 were asked to participate in this survey. A total of 12 consumers fitted with press-fit or screw-type osseointegration fixation between 07/2012 and 04/2019 responded to the survey, giving a return rate of 18%.
Experimental features	This specifically-designed survey was developed and administered by Queensland Artificial Limb Services (QALS), an Australian State government organization, as an integrated part of its continuous quality improvement procedure for assessing the provision of bone-anchored prosthesis. This 25-question survey was designed to assess change in quality of life experienced by QALS′ consumers before and after implantation of a press-fit or screw-type osseointegrated fixation when fitted with conventional socket-suspended and bone-anchored limb prostheses, respectively. The eligible consumers were asked to participate in this study over the phone by a QALS′ agent. Consumers could choose if they preferred receiving the survey by email or post.
Data source location	Brisbane, Queensland, Australia
Data accessibility	Data is with this article. Transparency data including the actual survey associated with this article can be found in the online version at https://data.mendeley.com/datasets/bkbxxmrhfh/1
Related research article	Frossard, L., Ferrada, L., Quincey, T., Burkett, B., and Berg, D., Development of a government continuous quality improvement procedure for assessing the provision of bone anchored limb prosthesis: A process re-design descriptive study. Canadian Prosthetics & Orthotics Journal, 2018. 1(2). p. 1–14 [1].

**Table:** 

**Value of the Data** • The survey data presented here provided an initial appraisal of the change in quality of life following surgical implantation of osseointegrated fixation, enabling direct skeletal attachment of the prosthesis, experienced by consumers supported by an Australian State government [1]. This specifically-designed survey focused on multiple facets of quality of life deriving essentially from safety and efficacy of the procedure as well as overall satisfaction with prosthesis [2], [3], [4]. This survey allowed comparing the baseline quality of life before the treatment when fitted with socket-suspended prosthesis with quality of life after treatment when fitted with bone-anchored prosthesis. This benchmark information could be used in future comparative studies or meta-analyses involving other cohorts of individuals fitted with socket-suspended or bone-anchored prostheses, respectively [5], [6], [7]. • This new insight into the quality of life reported by consumers fitted with bone-anchored prosthesis provided by a governmental organization can contribute to inform the design of specific and more advanced quality of life surveys that could be administered by other government organizations supporting provision of bone-anchored prosthesis. This information will be particularly valuable to those who have limited opportunities to administer standard generic health-related quality of life surveys (e.g., SF-36) as part of their continuous quality improvement of procedure [1]. • This quality of life data can also be valuable for researchers designing observational studies and clinical trials looking at the overall effects of particular interventions (e.g., design of osseointegrated fixation, effects of bone-anchored prosthesis components) on patients' satisfaction and quality of life. For instance, the magnitude of the difference between quality of life experienced with socket-suspended and bone-anchored prosthesis can informed the sample size required to achieve sufficient statistical power during the analytical planning stage.

## 1 Data

Table 1 presented the three levels of focus, actual question and type of answer for each of the 25 questions focusing on quality of life of consumers fitted with socket-suspended and bone-anchored prosthesis provided by Queensland Artificial Limb Services (QALS) before and after implantation of osseointegrated fixation.

**Table 1:** Three levels of focus, actual question and types of answer for each of the 25 questions in initial survey focusing on quality of life of consumers socket-suspended prosthesis (Items 1 to 7) and bone-anchored prosthesis (Items 8 to 24) provided by Queensland Artificial Limb Services before and after implantation of osseointegrated fixation, respectively.

Focus		Question		Answer
**1**	**Quality of life with socket-suspended prosthesis before treatment**			
**1.1**	**Efficacy**			
1.1.1	Function	Q8	Before undergoing Osseointegration did you use a socket prosthesis?	Dichotomous (Yes or no)
1.1.2	Function	Q9	How long did you use a socket prosthesis prior to having Osseointegration?	Open-ended (Enter number of years and months)
1.1.3	Function	Q10	How many hours per day were you able to wear the socket prosthetic limb?	Open-ended (Enter number of hours)
1.1.4	Function	Q11	Were you able to perform normal activities with a socket prosthesis?	Dichotomous (Yes or no)
**1.2**	**Experience**			
1.2.1	Satisfaction	Q12	Please indicate on the line below your level of quality of life with a socket prosthesis	Likert-type scale (O: Not Satisfied, 10: Very Satisfied)
**1.3**	**Knowledge**			
1.3.1	Motivation	Q2	Why did you decide to have Osseointegration?	Open-ended (Supply own answer)
1.3.2	Information	Q3	How did you hear about Osseointegration?	Open-ended (Supply own answer)
**2**	**Quality of life with bone-anchored prosthesis after treatment**			
**2.1**	**Surgery**			
2.1.1	Onset	Q1	When did you undergo the Osseointegration Surgery?	Open-ended (Enter date)
2.1.2	Satisfaction	Q7	Please indicate on the line below your initial level of satisfaction after your osseointegration surgery	Likert-type scale (O: Not Satisfied, 10: Very Satisfied)
**2.2**	**Safety**			
2.2.1	Infection	Q4	Did you experience any infections around your abutment exit point post-surgery?	Dichotomous (Yes or no)
2.2.2	Infection	Q5	If [your experienced infection around our abutment exit point post-surgery]– how long did you have infections for?	Open-ended (Enter number of days, weeks or months)
2.2.3	Infection	Q13	Have you developed any infections or irritation since the initial surgery?	Dichotomous (Yes or no)
**2.3**	**Efficacy**			
2.3.1	Function	Q6	How soon after the osseo surgery were you able to return to normal activities?	Open-ended (Enter number of days and weeks)
2.3.2	Function	Q14	Are you able to mobilise on an Osseointegrated Prosthesis?	Dichotomous (Yes or no)
2.3.3	Function	Q15	How long have you been mobilising with a Osseointegration Prosthesis?	Open-ended (Enter number of years and months)
2.3.4	Function	Q16	Does your Osseointegrated prosthesis function as it should?	Dichotomous (Yes or no)
2.3.5	Function	Q19	How many hours per day are you able to wear the Osseointegrated Prosthesis?	Open-ended (Enter number of hours)
2.3.6	Function	Q20	Would you like to be able to wear it more?	Dichotomous (Yes or no)
**2.4**	**Experience**			
2.4.1	Satisfaction	Q17	Are you satisfied with the componentry fitted to your Osseointegrated prosthesis?	Dichotomous (Yes or no)
2.4.2	Satisfaction	Q18	Overall, were you happy with your Osseointegration prosthesis?	Dichotomous (Yes or no)
2.4.3	Limitation	Q21	If [you like to be able to wear it more], what stops you from wearing it as much as you would like to?	Open-ended (Supply own answer)
2.4.4	Satisfaction	Q22	Does your Osseointegration Prosthesis support your life style needs?	Dichotomous (Yes or no)
2.4.5	Limitation	Q23	If [our Osseointegration Prosthesis support your life style does not support your lifestyle] – please state why	Open-ended (Supply own answer)
2.4.6	Satisfaction	Q24	Please indicate on the line below your level of quality of life with Osseointegration	Likert-type scale (O: Not Satisfied, 10: Very Satisfied)
**3**	**General comments**			
3.1	Comment	Q25	Any additional comments	Open-ended (Supply own answer)

Table 2 presented the case-mix profiles including demographics, amputation, access to care and funder information for the QALS' consumers fitted with bone-anchored prosthesis who were asked to participate in the study (N = 65) and responded (N = 12).

**Table 2:** Case-mix profiles including demographics, amputation, access to care and funder information for the Queensland Artificial Limb Services (QALS) consumers fitted with press-fit (N = 64) or screw-type (N = 1) osseointegrated fixation and bone-anchored prosthesis between 01/2011 and 06/2019 who were asked to participate in the study (N = 65) and responded (N = 12 including incomplete record for one respondent). PSP: Prosthetic Service Provider, DVA: Rehabilitation Appliance Program of the Department of Veteran Affairs, NDIS: National Disability Insurance Scheme.

	Overall population		Respondent population	
	(N = 65)		(N = 11)	
	Number	Mean ± SD	Number	Mean ± SD
**Demographics**				
Male	50 (77%)	–	10 (91%)	–
Female	15 (23%)	–	1 (9%)	–
Age (years)	65 (100%)	52 ± 13	11 (100%)	57 ± 12
Height (m)	58 (89%)	1.75 ± 0.10	10 (91%)	1.75 ± 0.08
Mass (kg)	62 (95%)	82.86 ± 17.29	11 (100%)	79.82 ± 17.92
**Amputation**				
**Timeline**				
Time since first amputation (years)	65 (100%)	20 ± 15	11 (100%)	17 ± 13
Time since first surgery for BAP (years)	64 (98%)	3 ± 1	11 (100%)	3 ± 1
**Cause**				
Trauma	44 (68%)	–	6 (55%)	–
Vascular insufficiency	9 (14%)	–	0 (0%)	–
Malignant neoplasm	6 (9%)	–	4 (36%)	–
**Level of amputation**				
Transfemoral	53 (82%)	–	9 (82%)	–
Transtibial	9 (14%)	–	2 (18%)	–
Through Knee	3 (5%)	–	0 (0%)	–
Hip disarticulation	1 (2%)	–	0 (0%)	–
**Number of amputations**				
Unilateral	58 (89%)	–	9 (82%)	–
Bilateral	5 (8%)	–	1 (9%)	–
Quadrilateral	2 (3%)	–	1 (9%)	–
**Access to prosthetic care**				
Distance-Residence to PSP (km)	60 (92%)	145 ± 212	11 (100%)	162 ± 248
Distance-Residence to QALS (km)	62 (95%)	364 ± 499	11 (100%)	369 ± 506
**Funder**				
QALS	38 (58%)	–	9 (82%)	–
DVA	8 (12%)	–	1 (9%)	–
NDIS	12 (18%)	–	1 (9%)	–

**Fig. 1:**
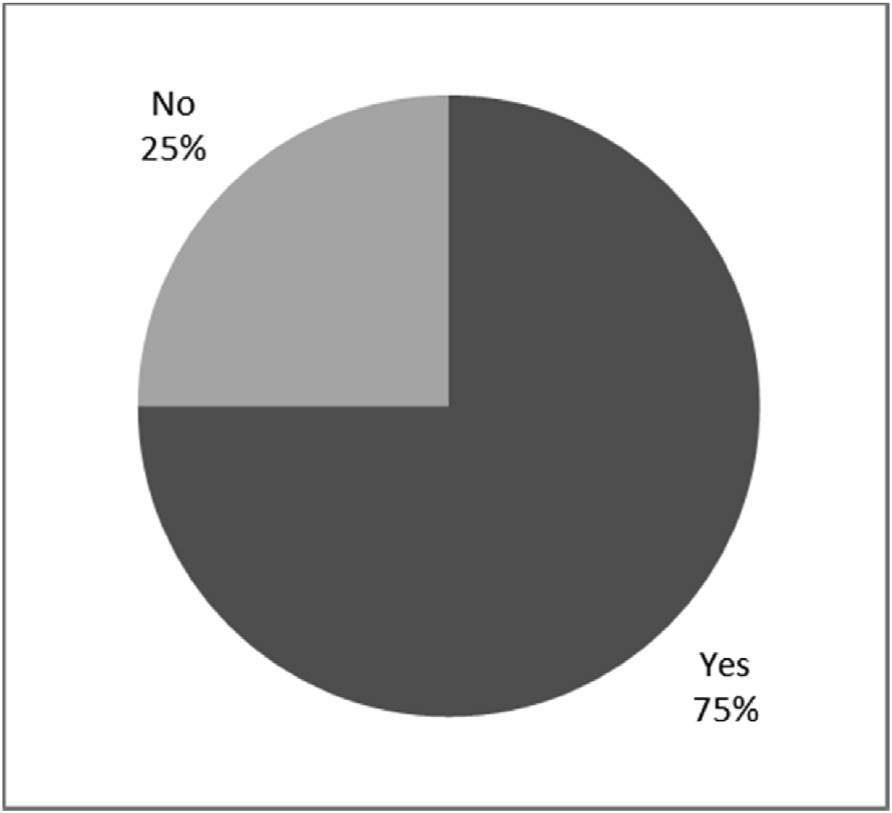
Outcomes of Q8 focusing on the capacity to use socket-suspended prosthesis before surgical implantation of the osseointegrated fixation (Q8: Before undergoing Osseointegration did you use a socket prosthesis?, Response rate: 100%).

**Fig. 2:**
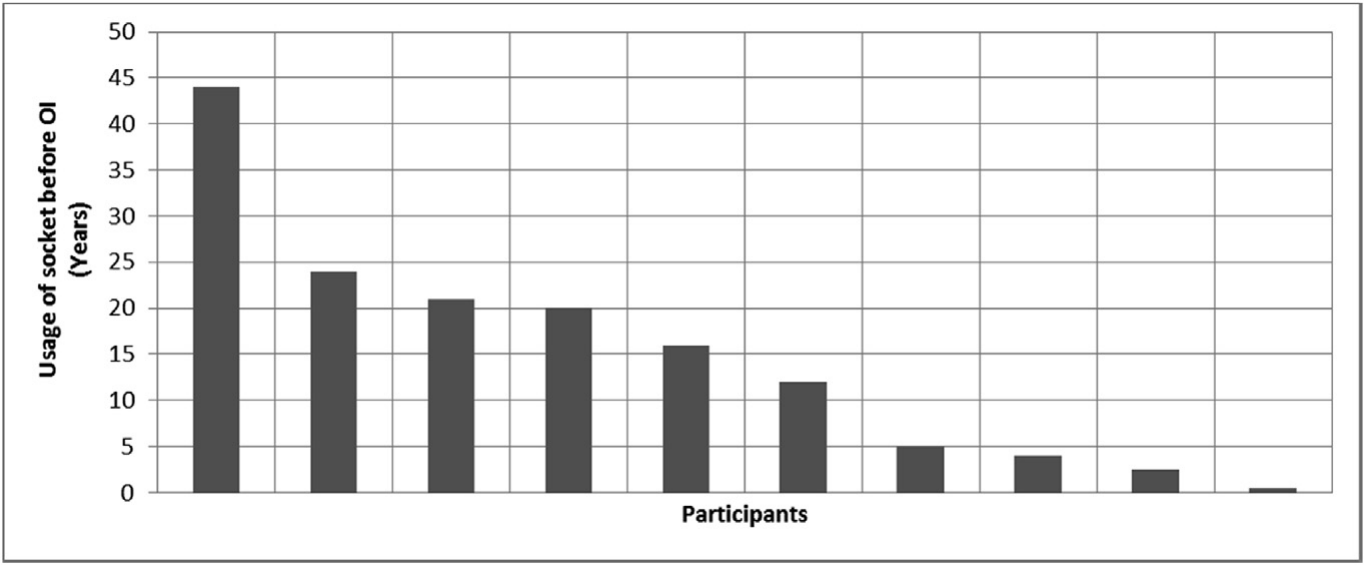
Outcomes of Q9 focusing on the duration of usage of socket-suspended prosthesis before surgical implantation of the osseointegrated fixation (Q9: How long did you use a socket prosthesis prior to having Osseointegration?, Response rate: 83%, Mean: 14.90 ± 13.25 years).

**Fig. 3:**
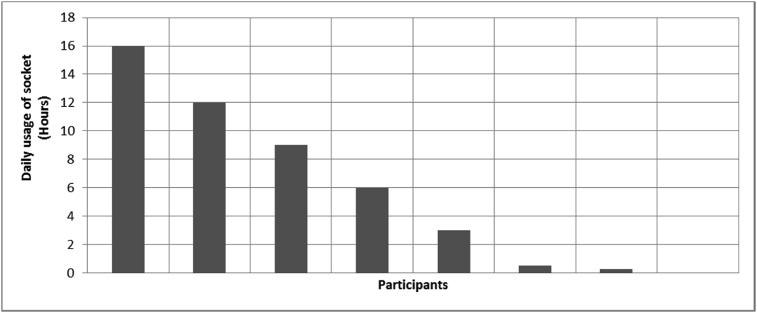
Outcomes of Q10 focusing on the daily ability to wear socket-suspended prosthesis before surgical implantation of the osseointegrated fixation (Q10: How many hours per day were you able to wear the socket prosthetic limb?, Response rate: 67%, Mean: 5.84 ± 6.01 hours).

**Fig. 4:**
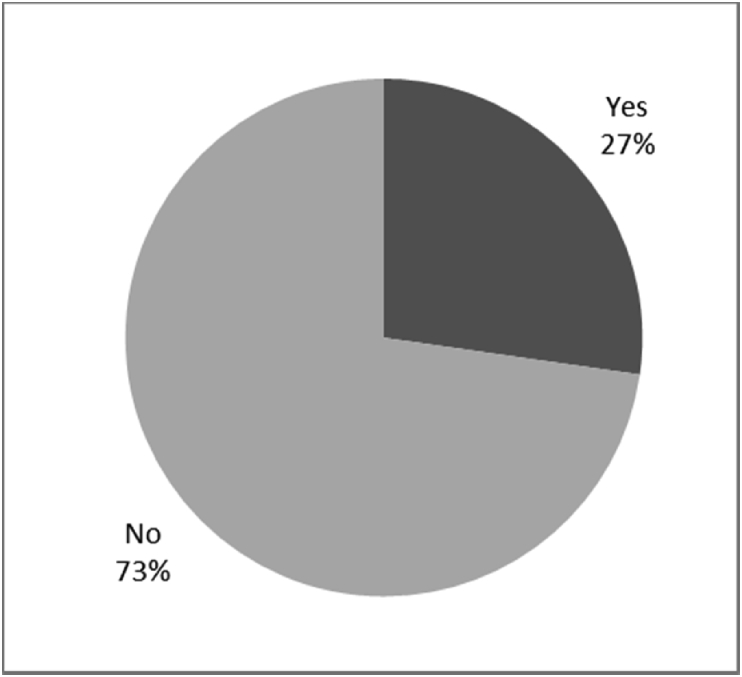
Outcomes of Q11 focusing on the ability to perform daily activities with socket-suspended prosthesis before surgical implantation of the osseointegrated fixation (Q11: Were you able to perform normal activities with a socket prosthesis?, Response rate: 92%).

**Fig. 5:**
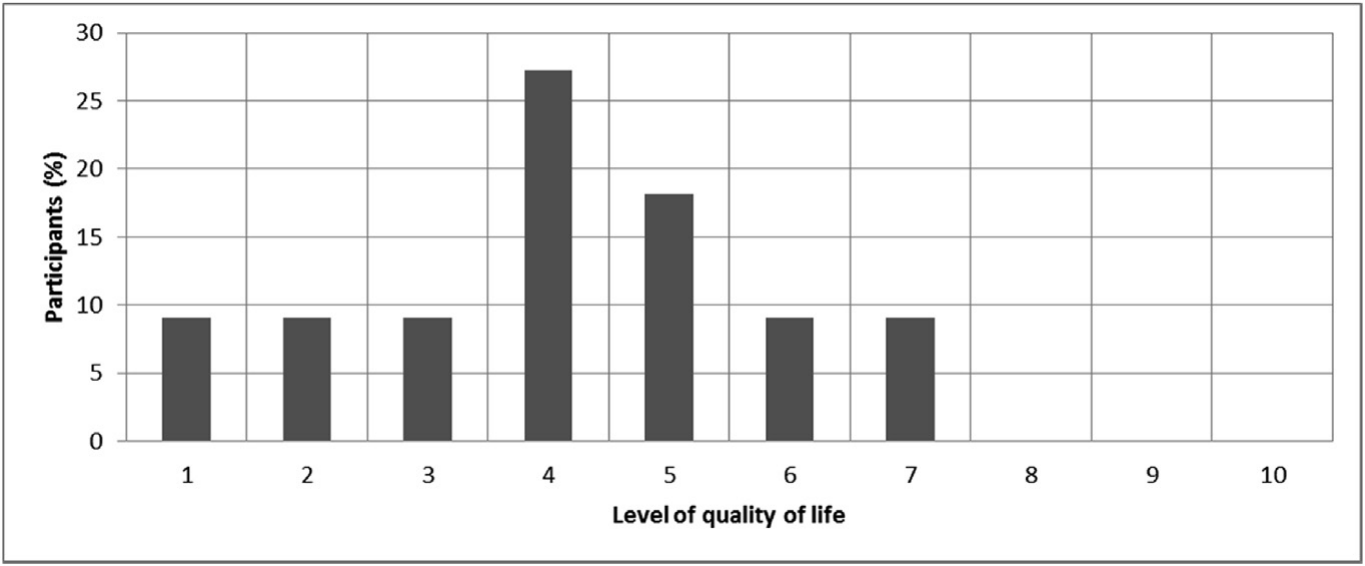
Outcomes of Q12 focusing on the percentage of participants in each level of quality of life with socket-suspended prosthesis before surgical implantation of the osseointegrated fixation ranging between 0 (not satisfied) and 10 (very satisfied) (Q12: Please indicate on the line below your level of quality of life with a socket prosthesis, Response rate: 92%, Mean: 3.73 ± 2.10).

**Fig. 6:**
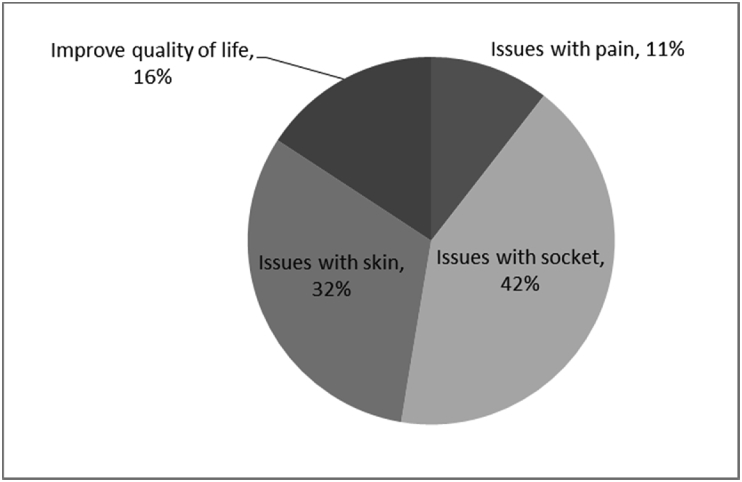
Outcomes of Q2 focusing on the motivations for choosing surgical implantation of the osseointegrated fixation (Q2: Why did you decide to have Osseointegration?, Response rate: 100%).

**Fig. 7:**
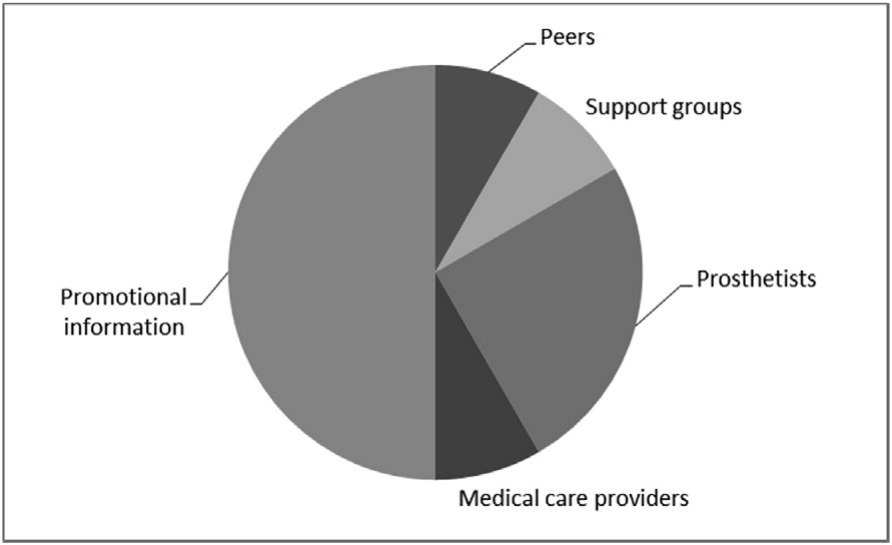
Outcomes of Q3 focusing on the source of information found about surgical procedure for implantation of the osseointegrated fixation (Q3: How did you hear about Osseointegration?, Response rate: 100%).

Fig. 1, Fig. 2, Fig. 3, Fig. 4, Fig. 5, Fig. 6, Fig. 7 provided the baseline outcomes for the seven questions related to the quality of life of QALS' consumers provided with socket-suspended prosthesis before implantation of osseointegrated fixation focusing on efficacy (i.e., Q8, Q9, Q10, Q11), experience (i.e., Q12) and knowledge of the osseointegration treatment (i.e., Q2, Q3).

Fig. 8, Fig. 9, Fig. 10, Fig. 11, Fig. 12, Fig. 13, Fig. 14, Fig. 15, Fig. 16, Fig. 17, Fig. 18, Fig. 19, Fig. 20, Fig. 21, Fig. 22, Fig. 23, Fig. 24 provided the outcomes for the 17 questions related to the quality of life of QALS' consumers provided with bone-anchored prosthesis after implantation of osseointegrated fixation focusing on surgery (i.e., Q1, Q7), safety and harms (i.e., Q4, Q5, Q13), efficacy and benefits (i.e., Q6, Q14, Q15, Q16, Q19, Q20) and overall experience (i.e., Q17, Q18, Q21, Q22, Q23, Q24).

**Fig. 8:**
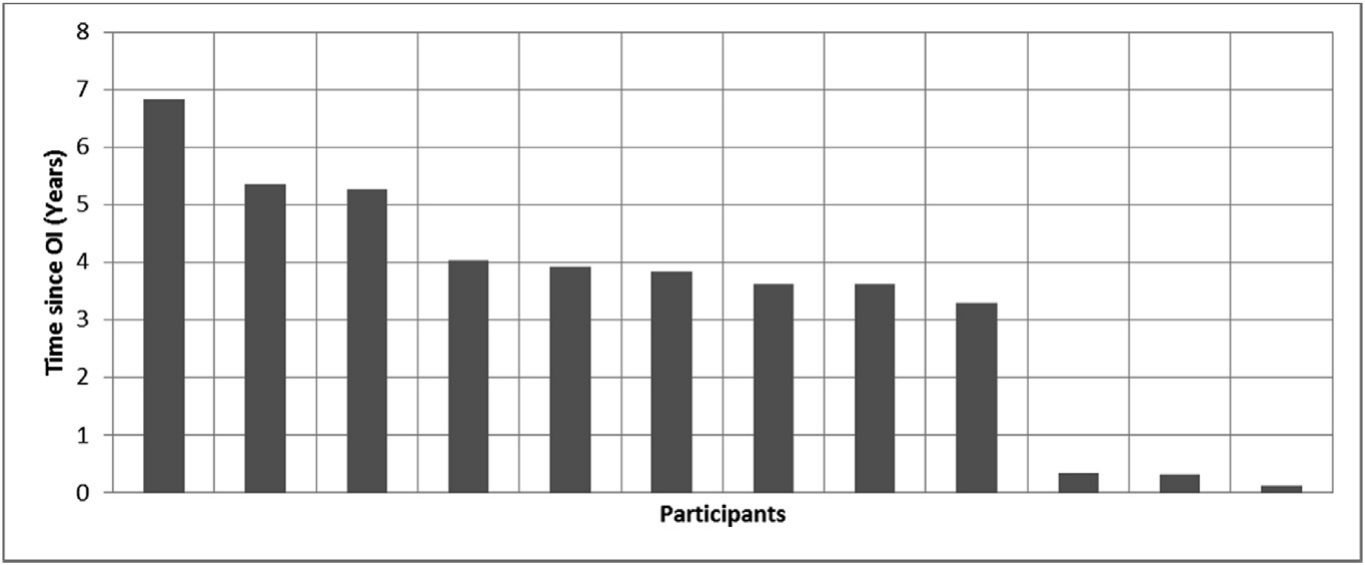
Outcomes of Q1 focusing on the time since the surgical implantation of the osseointegrated fixation (Q1: When did you undergo the Osseointegration Surgery?, Response rate: 100%, Mean: 3.37 ± 2.12 years).

**Fig. 9:**
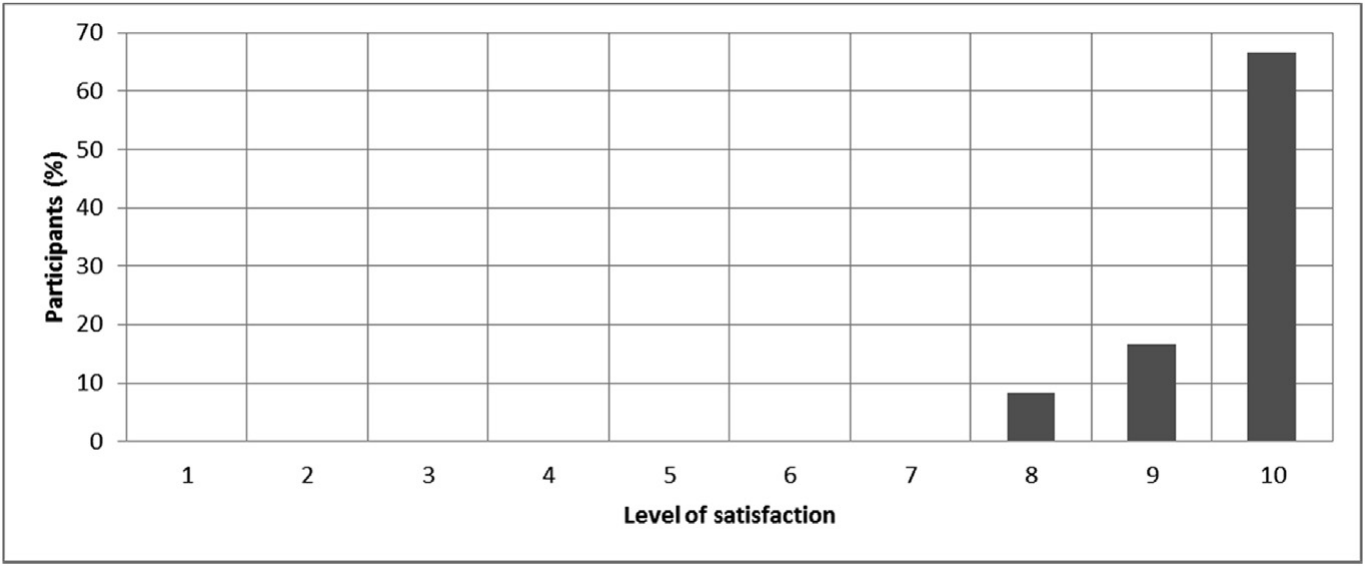
Outcomes of Q7 focusing on the percentage of participants in each level of satisfaction after surgical implantation of the osseointegrated fixation ranging between 0 (not satisfied) and 10 (very satisfied) (Q7: Please indicate on the line below your initial level of satisfaction after your osseointegration surgery, Response rate: 100%, Mean: 9.54 ± 0.72).

**Fig. 10:**
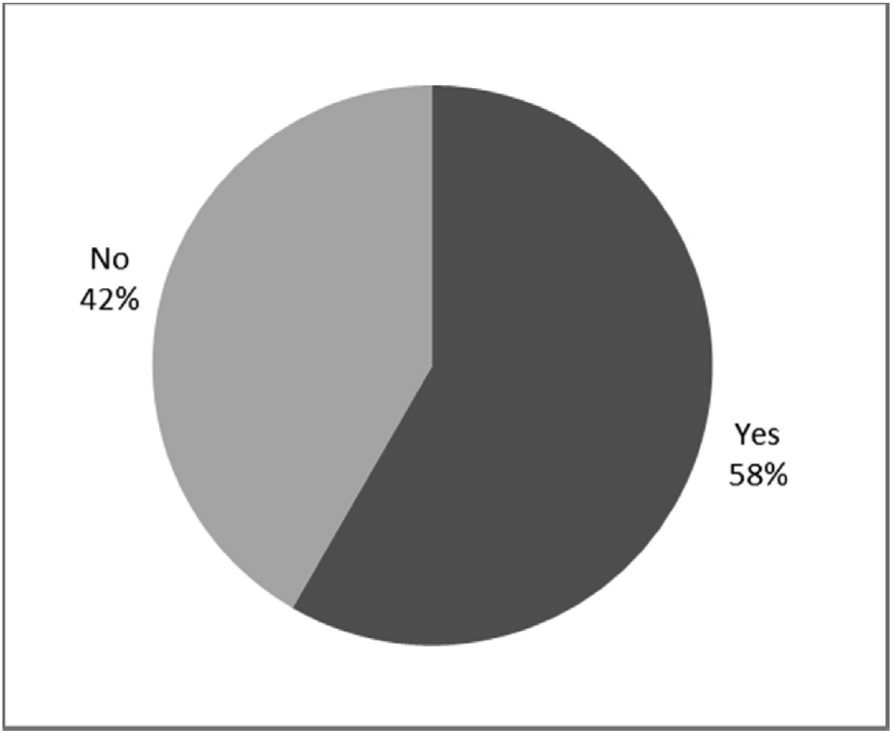
Outcomes of Q4 focusing on the infection experienced after the surgical implantation of the osseointegrated fixation (Q4: Did you experience any infections around your abutment exit point post-surgery?, Response rate: 100%).

**Fig. 11:**
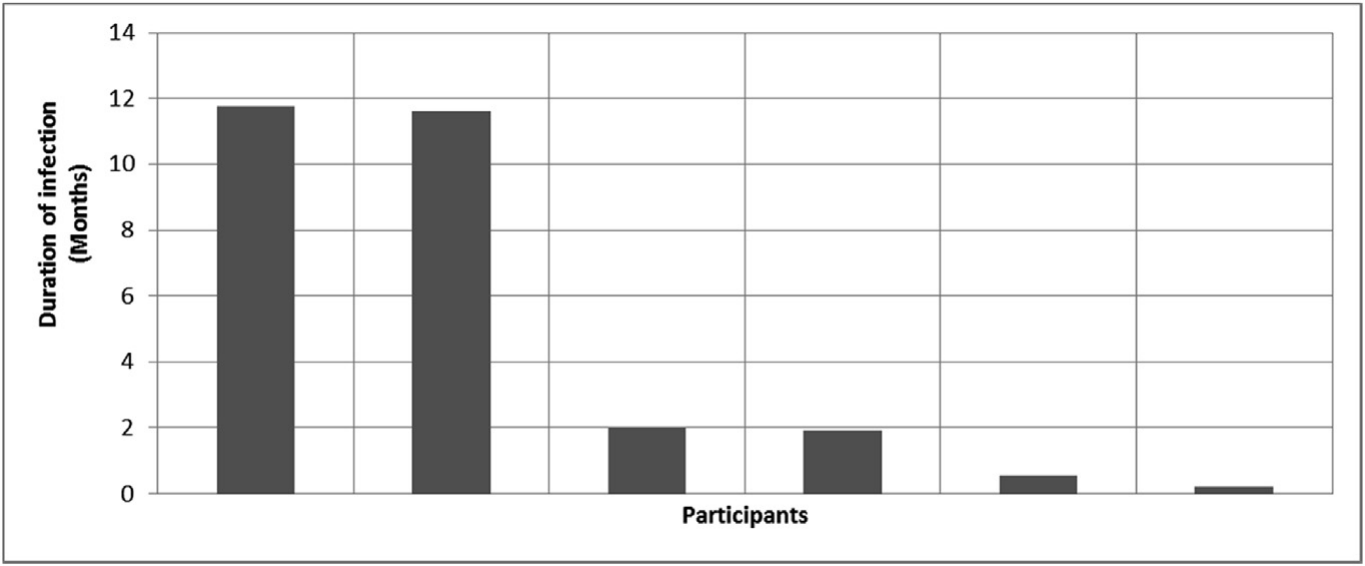
Outcomes of Q5 focusing on the duration of infection after surgical implantation of the osseointegrated fixation (Q5: If [your experienced infection around our abutment exit point post-surgery]– how long did you have infections for?, Response rate: 50%, Mean: 4.68 ± 5.48 months).

**Fig. 12:**
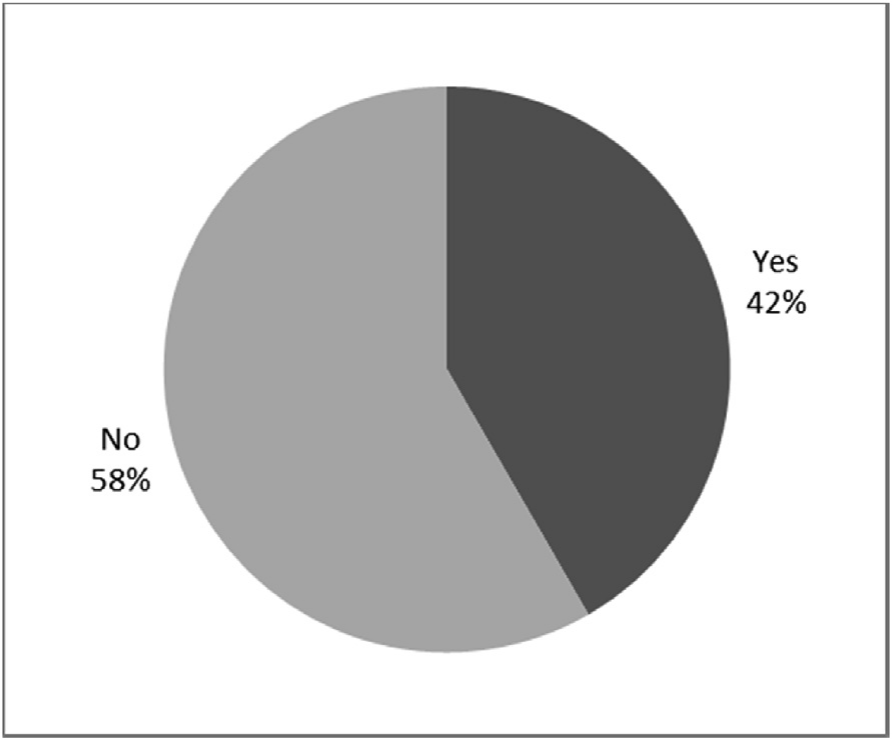
Outcomes of Q13 focusing on the incidence of infection or irrational of stoma since surgical implantation of the osseointegrated fixation (Q13-Have you developed any infections or irritation since the initial surgery? Response rate: 100%).

**Fig. 13:**
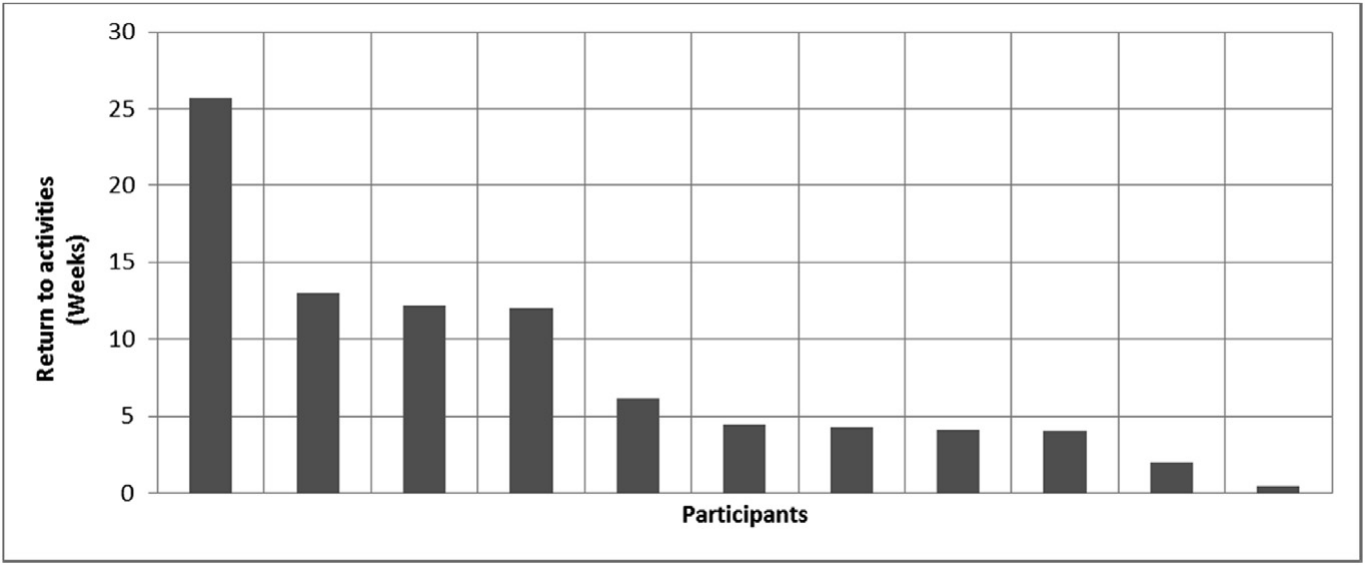
Outcomes of Q6 focusing on the lapse between surgical implantation of the osseointegrated fixation and the return to normal activities (Q6: How soon after the osseo surgery were you able to return to normal activities?, Response rate: 92%, Mean: 8.03 ± 7.25 weeks).

**Fig. 14:**
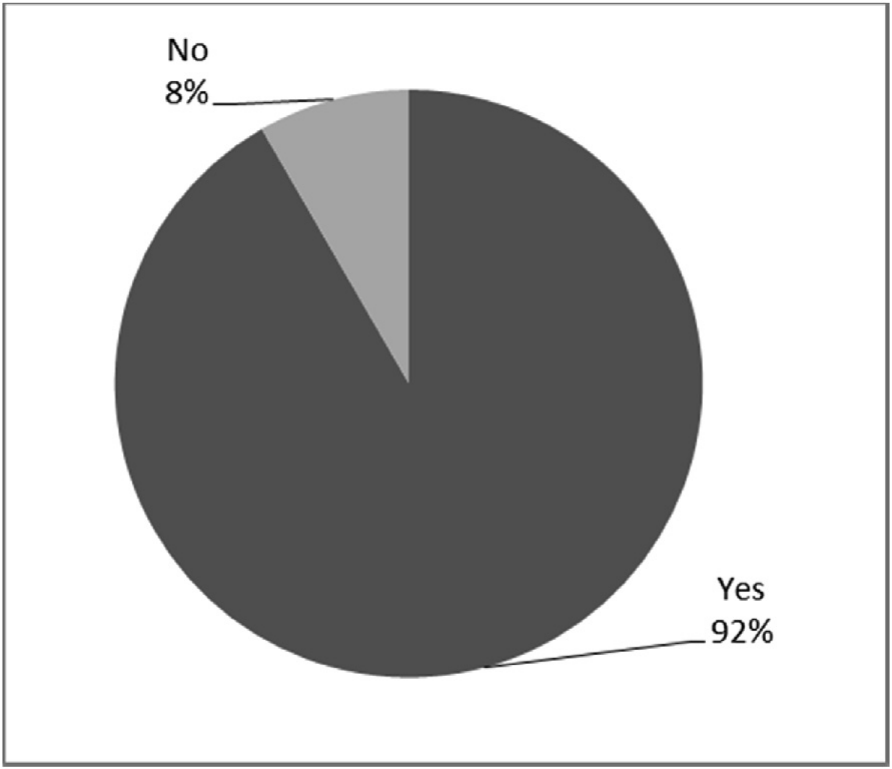
Outcomes of Q14 focusing on the ability to use bone-anchored prosthesis since surgical implantation of the osseointegrated fixation (Q14: Are you able to mobilise on an Osseointegrated Prosthesis?, Response rate: 100%).

**Fig. 15:**
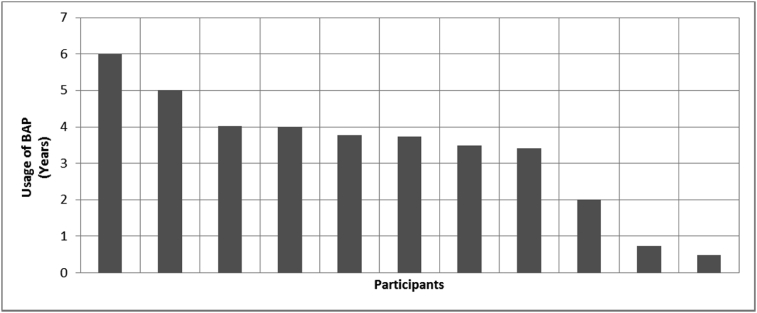
Outcomes of Q15 focusing on the ability to wear bone-anchored prosthesis since surgical implantation of the osseointegrated fixation (Q15: How long have you been mobilising with a Osseointegration Prosthesis?, Response rate: 92%, Mean: 3.33 ± 1.67 years).

**Fig. 16:**
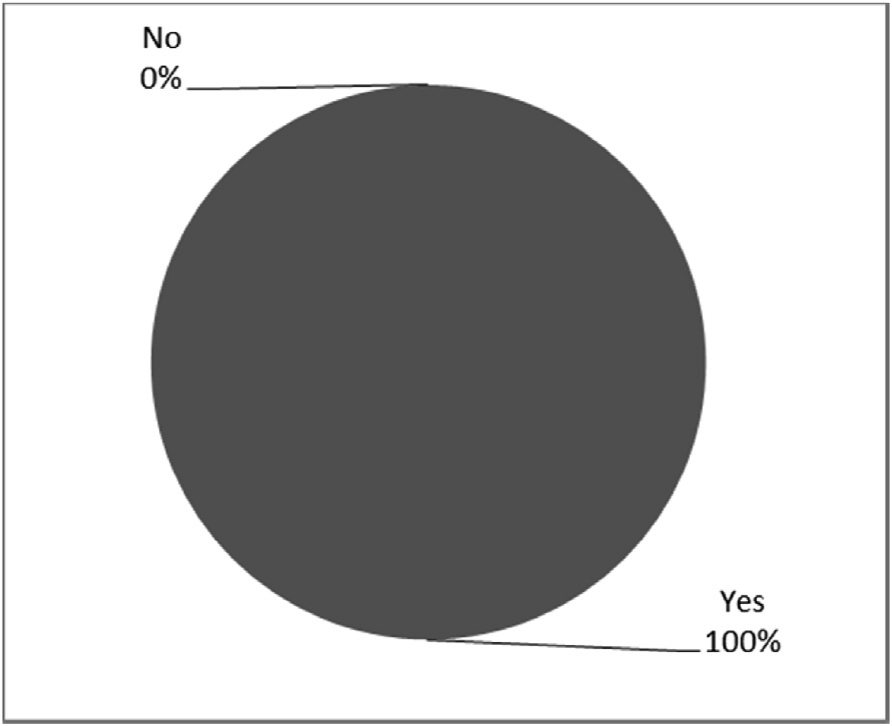
Outcomes of Q16 focusing on the functionality of bone-anchored prosthesis since surgical implantation of the osseointegrated fixation (Q16: Does your Osseointegrated prosthesis function as it should?, Response rate: 92%).

**Fig. 17:**
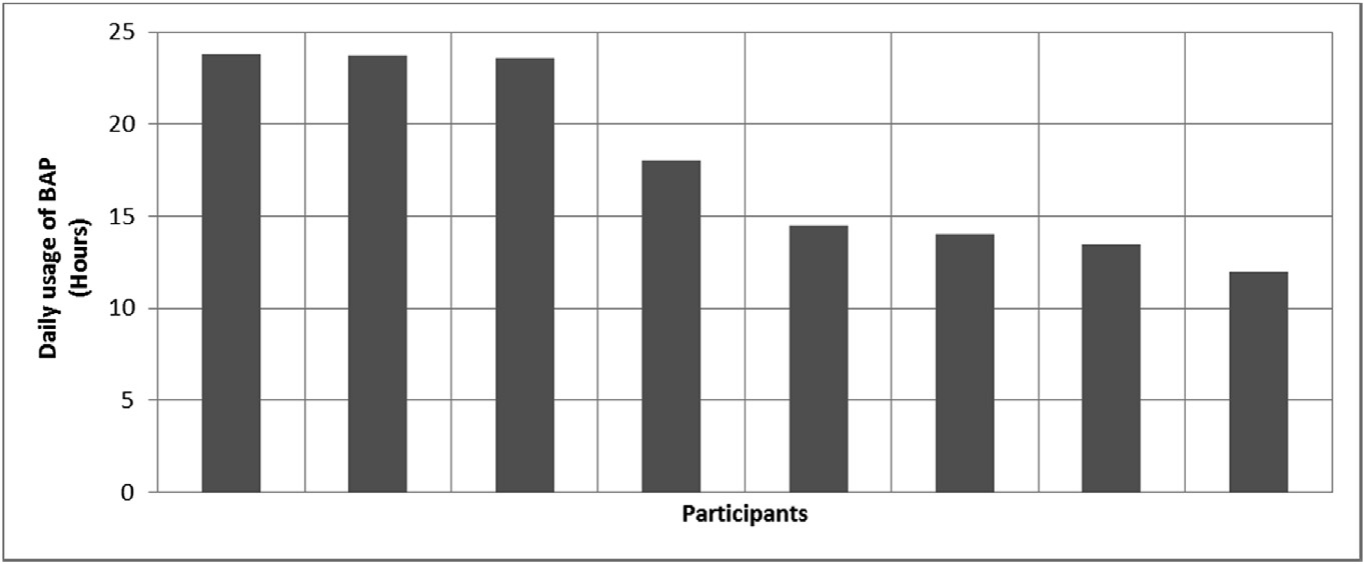
Outcomes of Q19 focusing on the daily ability to wear bone-anchored prosthesis since surgical implantation of the osseointegrated fixation (Q19-How many hours per day are you able to wear the Osseointegrated Prosthesis? Response rate: 75%, Mean: 17.89 ± 5.10 hours).

**Fig. 18:**
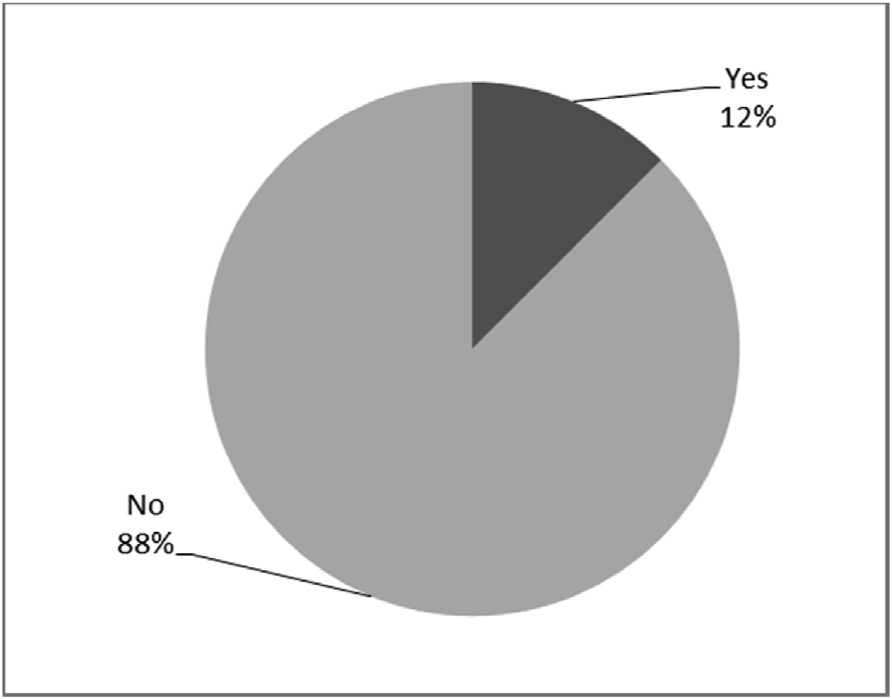
Outcomes of Q20 focusing on the aspiration to increase usage of bone-anchored prosthesis since surgical implantation of the osseointegrated fixation (Q20-Would you like to be able to wear it more? Response rate: 67%).

**Fig. 19:**
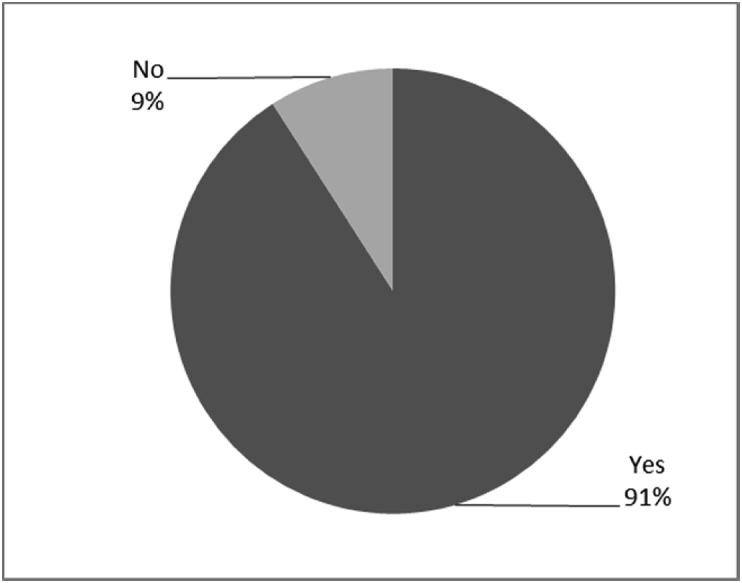
Outcomes of Q17 focusing on the satisfaction with components fitted in bone-anchored prosthesis since surgical implantation of the osseointegrated fixation (Q17: Are you satisfied with the componentry fitted to your Osseointegrated prosthesis?, Response rate: 92%).

**Fig. 20:**
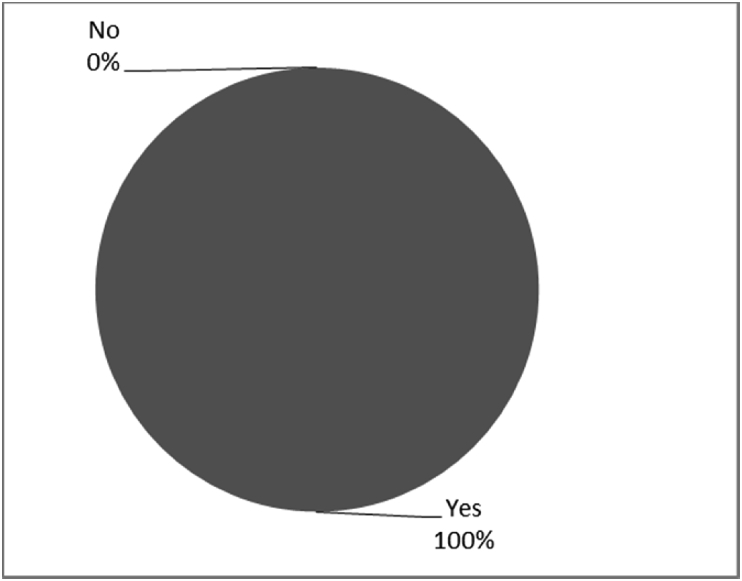
Outcomes of Q18 focusing on the satisfaction with bone-anchored prosthesis since surgical implantation of the osseointegrated fixation (Q18: Overall, were you happy with your Osseointegration prosthesis?, Response rate: 92%).

**Fig. 21:**
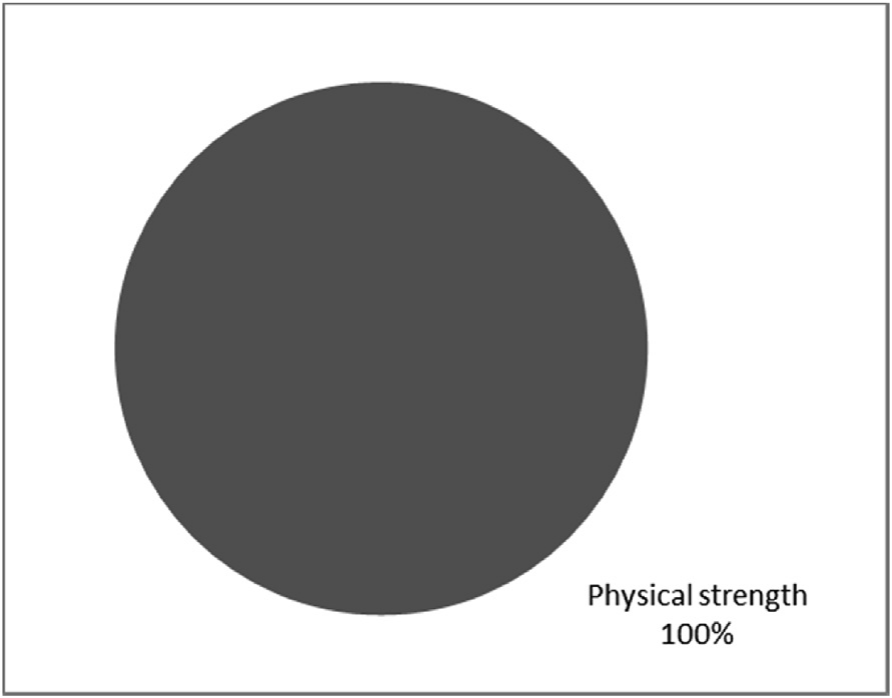
Outcomes of Q21 focusing on the description of possible limitations to the use of bone-anchored prosthesis since surgical implantation of the osseointegrated fixation (Q21: if [you like to be able to wear it more], what stops you from wearing it as much as you would like to?, Response rate: 8%).

**Fig. 22:**
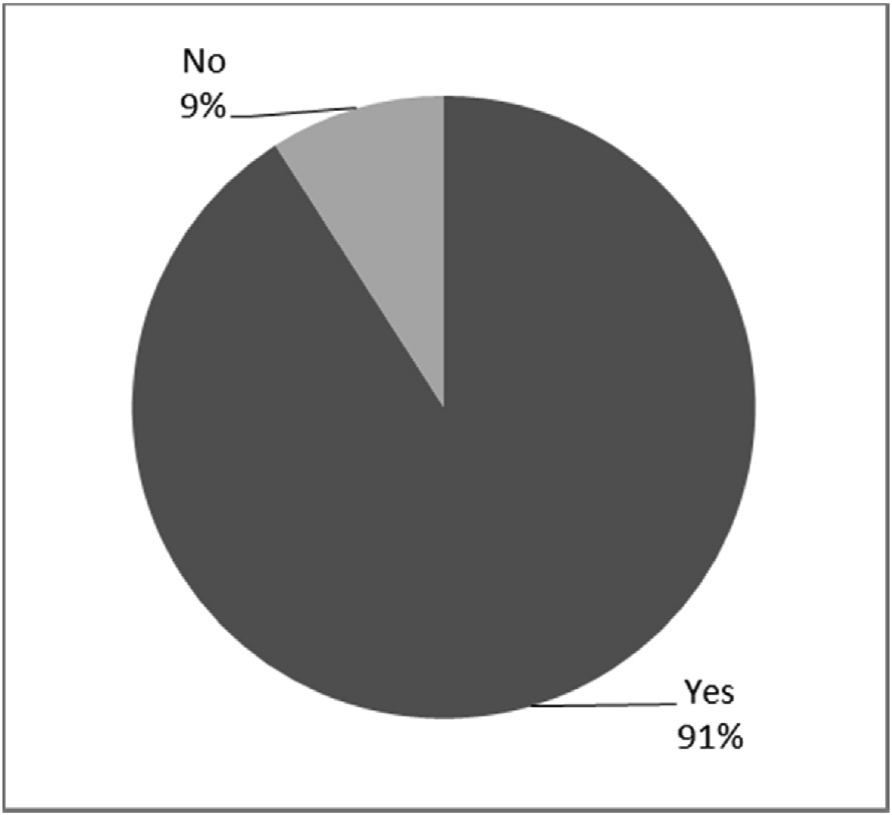
Outcomes of Q22 focusing on the ability to support the lifestyle using bone-anchored prosthesis since surgical implantation of the osseointegrated fixation (Q22-Does your Osseointegration Prosthesis support your life style needs? Response rate: 92%).

**Fig. 23:**
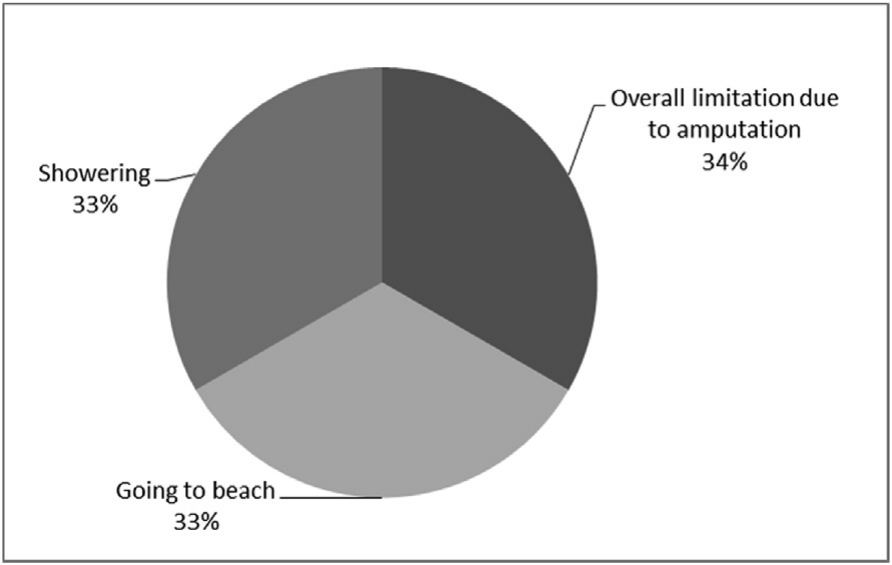
Outcomes of Q23 focusing on the description of limitations to support the lifestyle using bone-anchored prosthesis since surgical implantation of the osseointegrated fixation (Q23: If [our Osseointegration Prosthesis support your life style does not support your lifestyle] – please state why, Response rate: 25%).

**Fig. 24:**
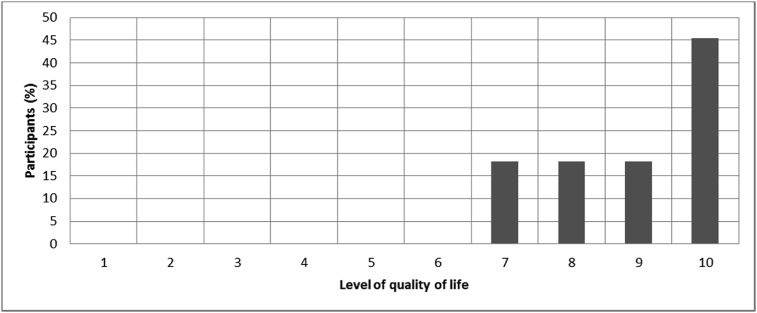
Outcomes of Q24 focusing on the percentage of participants in each level of quality of life with bone-anchored prosthesis since surgical implantation of the osseointegrated fixation ranging between 0 (not satisfied) and 10 (very satisfied) (Q24-Please indicate on the line below your level of quality of life with Osseointegration Response rate: 92%, Mean: 8.91 ± 1.22).

**Fig. 25:**
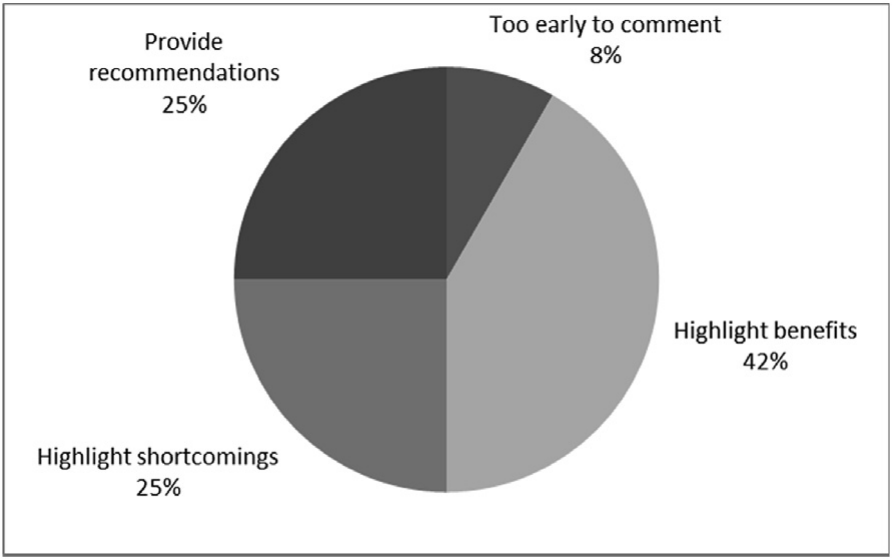
Outcomes of Q25 focusing on the percentage of additional comments in given categories (Q25: Any additional comments, Response rate: 100%).

Fig. 25 showed the general comments provided by consumers (i.e., Q25).

## 2 Design of survey

The initial survey focusing on quality of life of consumers with socket-suspended prosthesis or bone-anchored prosthesis provided by Queensland Artificial Limb Services included 25 questions, as described in Table 1.

## 3 Population

The Queensland Artificial Limb Services asked 65 consumers with osseointegrated fixation and bone-anchored prosthesis to complete the survey as presented in Table 2.

## 4 Quality of life with socket-suspended prosthesis before treatment

The baseline outcomes for the seven questions related to the quality of life of QALS’ consumers provided with socket-suspended prosthesis before implantation of osseointegrated fixation focusing on efficacy (i.e., Q8, Q9, Q10, Q11), experience (i.e., Q12) and knowledge of the osseointegration treatment (i.e., Q2, Q3) are presented in Fig. 1, Fig. 2, Fig. 3, Fig. 4, Fig. 5, Fig. 6, Fig. 7.

## 5 Quality of life with bone-anchored prosthesis after treatment

The outcomes for the questions related to the quality of life of QALS’ consumers provided with bone-anchored prosthesis after implantation of osseointegrated fixation focusing on surgery (i.e., Q1, Q7), safety and harms (i.e., Q4, Q5, Q13), efficacy and benefits (i.e., Q6, Q14, Q15, Q16, Q19, Q20) and overall experience (i.e., Q17, Q18, Q21, Q22, Q23, Q24) are presented in Fig. 8, Fig. 9, Fig. 10, Fig. 11, Fig. 12, Fig. 13, Fig. 14, Fig. 15, Fig. 16, Fig. 17, Fig. 18, Fig. 19, Fig. 20, Fig. 21, Fig. 22, Fig. 23, Fig. 24.

## 6 General comments

The general comments provided by consumers (i.e., Q25) are summarized in Fig. 25.

### 6.1 Experimental design, materials, and methods

#### 6.1.1 Participants

This study involved all of 65 QALS′ consumers fitted with at least one bone-anchored prosthesis after implantation of press-fit (N = 64) or screw-type (N = 1) osseointegration fixation between 01/2011 and 06/2019. This cohort represented circa 16% and 7% of existing population estimated at 400 in Australia and 950 worldwide, respectively [1]. A total of 12 out of 65 consumers fitted with bone-anchored prosthesis between 07/2012 and 04/2019 responded to the survey, giving a return rate of 18%. The individual question's response rate corresponded to the number of responses for a given question over 12 respondents.

#### 6.1.2 Survey

This specifically-designed survey data on the quality of life was administered by Queensland Artificial Limb Services (QALS), an Australian State government organization, as an integrated part of its continuous quality improvement procedure for assessing the provision of bone-anchored prosthesis [1], [8], [9], [10]. This survey was designed to assess change in quality of life experienced by QALS's consumers before and after implantation of a press-fit or screw-type osseointegrated fixation when fitted with conventional socket-suspended and bone-anchored limb prosthesis, respectively [3], [4], [11].

First, participants were required to indicate their name, address, date of birth and email. Then, participants answered 25 questions organized around the three following sections:

• 7 (28%) questions about “Osseointegration Surgery Details” (i.e., Q1 – 7),
• 5 (20%) questions about “Pre-Osseointegration Surgery” (i.e., Q8 – 12),
• 13 (52%) questions about “Post-Surgery Osseointegration” (i.e., Q13 – 25).

The 65 eligible consumers were asked to participate in this study over the phone by a QALS' agent. Consumers could choose if they preferred receiving the survey by email or by post with pre-paid return envelope.

#### 6.1.3 Data mapping

Analysis of the survey data consisted in extracting information for:

• 7 (28%) questions providing baseline outcomes that related to the quality of life of QALS′ consumers fitted with socket-suspended prosthesis before implantation of osseointegrated fixation including:
  ○ 4 (16%) questions focusing on efficacy, particularly the level of function (i.e., Q8, Q9, Q10, Q11),
  ○ 1 (4%) question focusing on experience, particularly the level of satisfaction (i.e., Q12),
  ○ 3 (12%) questions focusing on knowledge of the osseointegration treatment (i.e., Q2, Q3), particularly the motivation for considering the procedure and the sources of information considered where promotional information included TV and social media.
• 17 (68%) questions assessing the quality of life of QALS′ consumers fitted with bone-anchored prosthesis after implantation of osseointegrated fixation including:
  ○ 2 (8%) questions focusing on surgery (i.e., Q1, Q7), particularly the time of the surgery and the level of satisfaction with the procedure,
  ○ 2 (8%) questions focusing on safety or harms (i.e., Q5, Q13, Q4), particularly the occurrence of infection [12], [13],
• 7 (28%) questions providing baseline outcomes that related to the quality of life of QALS′ consumers fitted with socket-suspended prosthesis before implantation of osseointegrated fixation including:
  ○ 4 (16%) questions focusing on efficacy, particularly the level of function (i.e., Q8, Q9, Q10, Q11),
  ○ 1 (4%) question focusing on experience, particularly the level of satisfaction (i.e., Q12),
  ○ 3 (12%) questions focusing on knowledge of the osseointegration treatment (i.e., Q2, Q3), particularly the motivation for considering the procedure and the sources of information considered where promotional information included TV and social media.
• 17 (68%) questions assessing the quality of life of QALS′ consumers fitted with bone-anchored prosthesis after implantation of osseointegrated fixation including:
  ○ 2 (8%) questions focusing on surgery (i.e., Q1, Q7), particularly the time of the surgery and the level of satisfaction with the procedure,
  ○ 2 (8%) questions focusing on safety or harms (i.e., Q13, Q4, Q5), particularly the occurrence of infection [12], [13],
  ○ 6 (24%) questions focusing on efficacy or benefits (i.e., Q6, Q14, Q15, Q16, Q19, Q20), particularly the level of function [14], [15],
  ○ 6 (24%) questions focusing on overall experience (i.e., Q17, Q18, Q21, Q22, Q23, Q24), particularly the limitations and level of satisfaction.
• 1 (4%) general comments provided by consumers focusing on limitation of their observation time as well recommendations, benefits and shortcomings of the treatment (i.e., Q25).

Answers to the 10 (40%) dichotomous questions (i.e., Yes or no) were expressed in percentage of individual responses (i.e., Q4, Q8, Q11, Q13, Q14, Q16, Q17, Q18, Q20, Q22).

Answers to the 3 (12%) Likert-type scale questions were expressed as percentage of participants in each of the 10 levels between 0 for “not satisfied” and 10 for “very satisfied” (i.e., Q7, Q12, Q24).

Answers to the 12 (48%) open-ended questions were coded accordingly to the recurrence of themes in the replies (i.e., Q1, Q2, Q3, Q5, Q6, Q9, Q10, Q15, Q19, Q21, Q23, Q25).

○ 6 (24%) questions focusing on overall experience (i.e., Q21, Q23, Q17, Q18, Q22, Q24), particularly the limitations and level of satisfaction.

• 1 (4%) general comments provided by consumers focusing on limitation of their observation time as well recommendations, benefits and shortcomings of the treatment (i.e., Q25).

Answers to the 10 (40%) dichotomous questions (i.e., Yes or no) were expressed in percentage of individual responses (i.e., Q4, Q8, Q11, Q13, Q14, Q16, Q17, Q18, Q20, Q22).

Answers to the 3 (12%) Likert-type scale questions were expressed as percentage of participants in each of the 10 levels between 0 for “not satisfied” and 10 for “very satisfied” (i.e., Q7, Q12, Q24).

Answers to the 12 (48%) open-ended questions were coded accordingly to the recurrence of themes in the replies (i.e., Q1, Q2, Q3, Q5, Q6, Q9, Q10, Q15, Q19, Q21, Q23, Q25).

## 7 Data analysis

Only aggregated data was presented in this study. Exploration of more detailed analysis revealed that proportionate and disproportionate stratification sampling were unattainable given the diversity of case-mix and the small number of respondents.
