# Supplementary material for: Survey data on the quality of life of consumers fitted with osseointegrated fixation and bone-anchored limb prostheses provided by government organization
Source: Data Brief. 2019 Sep 17;26:104536. doi: 10.1016/j.dib.2019.104536 (PMC6811965; doi:10.1016/j.dib.2019.104536)
Supplement: Multimedia component 1 [file mmc1.pdf]

Initial Quality of Life Survey	This survey has been sent to you by the Queensland Artificial Limb Service (QALS) to monitor the service delivery and quality of life for people with an amputation who have undergone Osseointegration (Osseo/OI).

«GivenName» «Surname»
«Address»
«Suburb» «State» «PostCode»

Date of Birth: «DOB»
Email: «Email» ____________________
I would prefer future survey to be sent by:

Post ☐ Email ☐

Osseointegration Surgery Details:

1. When did you undergo the Osseointegration Surgery? ________________________ Day /Month /Year
2. Why did you decide to have Osseointegration? ____

   ____
3. How did you hear about Osseointegration? ____
4. Did you experience any infections around your abutment exit point post-surgery .... YES / NO
5. If YES – how long did you have infections for? ____________________ Days / Weeks/ Months
6. How soon after the osseo surgery were you able to return to normal activities? __________ Days /Weeks
7. Please indicate on the line below your initial level of satisfaction after your osseointegration surgery:

0	1	2	3	4	5	6	7	8	9	10
Not Satisfied										Very Satisfied

Pre-Osseointegration Surgery:

8. Before undergoing Osseointegration did you use a socket prosthesis? .... YES / NO
9. How long did you use a socket prosthesis prior to having Osseointegration? ____ Years ______ Months
10. How many hours per day were you able to wear the socket prosthetic limb? ____________________
11. Were you able to perform normal activities with a socket prosthesis? .... YES / NO
12. Please indicate on the line below your level of quality of life with a socket prosthesis:

0	1	2	3	4	5	6	7	8	9	10
Not Satisfied										Very Satisfied

Post-Surgery Osseointegration

13. Have you developed any infections or irritation since the initial surgery?.................................... YES /NO

14. Are you able to mobilise on an Osseointegrated Prosthesis? .... YES /NO

15. How long have you been mobilising with a Osseointegration Prosthesis? _______Years _______Months

16. Does your Osseointegrated prosthesis function as it should?.. YES / NO

17. Are you satisfied with the componentry fitted to your Osseointegrated prosthesis?..................... YES /NO

18. Overall, were you happy with your Osseointegration prosthesis? .... YES /NO

19. How many hours per day are you able to wear the Osseointegrated Prosthesis? ___________________

20. Would you like to be able to wear it more? .... YES /NO

21. If so, what stops you from wearing it as much as you would like to? _______________________________

____

22. Does your Osseointegration Prosthesis support your life style needs? .... YES /NO

23. If NO – please state why: ____

____

24. Please indicate on the line below your level of quality of life with Osseointegration:

____

0 1 2 3 4 5 6 7 8 9 10

Not Satisfied Very Satisfied

25. Any additional comments ____

____

____

____

____

____

The Queensland Artificial Limb Service thanks you for your feedback. Once the form is completed, please return it in the envelope provided or to the following addresses:
